# Kinetic Mechanisms and Emissions Investigation of Torrefied Pine Sawdust Utilized as Solid Fuel by Isothermal and Non-Isothermal Experiments

**DOI:** 10.3390/ma15238650

**Published:** 2022-12-04

**Authors:** Xiaorui Liu, Dong Li, Jiamin Yang, Longji Yuan

**Affiliations:** 1 School of Mine, China University of Mining and Technology, Xuzhou 221116, China; 2 School of Low-Carbon Energy and Power Engineering, China University of Mining and Technology, Xuzhou 221116, China

**Keywords:** biomass torrefaction, combustion mechanism, kinetics, emissions

## Abstract

This study comprehensively investigated the utilization of torrefied pine sawdust (PS) as solid fuels, involving the characterization of torrefied PS properties, the investigation of combustion behaviors and kinetic mechanisms by non-isothermal experiments, and the evaluation of emissions during isothermal experiments. Results show that torrefaction significantly improved the quality of the solids. The upgradation of torrefied PS properties then further enhanced its combustion performance. For the kinetics mechanisms, degradation mechanisms and diffusion mechanisms were respectively determined for the volatile combustion and the char combustion by using both Coats–Redfern (CR) and Freeman–Carroll (FC) methods. Further, after torrefaction, the emission of NO for volatile combustion reduced while it increased for char combustion. An inverse relationship was found between the conversion of fuel-N to NO and the nitrogen content in the torrefied samples. This study provided comprehensive insights for considering torrefaction as a pretreatment technique for PS utilization as a solid fuel.

## 1. Introduction

With the declaration of peak carbon emissions by 2030 and carbon neutral by 2060, the utilization of renewable energy sources, such as solar, wind, and biomass energy, etc., became increasingly important in China. As the only carbon-based material among these renewable energy sources, biomass has the ability to be converted into gases, liquids, chemicals and solid materials with zero CO_2_ emissions through its life cycle [1] due to which it is considered to be an indispensable material to partially substitute the consumption of traditional fossil fuels [2]. Nowadays, the common technologies for biomass utilization are thermochemical and biological conversion [3], wherein thermochemical conversion, including combustion, pyrolysis, gasification, etc., is the most efficient [1]. However, there are still challenges for biomass large scale utilization due to its high moisture, low energy density, and hydrophilia, as well as the high milling and transportation costs [4,5].

Within this context, various pretreatment technologies have been developed by researchers. Compared with pyrolysis and hydrothermal carbonization treatments, torrefaction which is actually a mild pyrolysis process is of less energy consumption and costs [6]. It consumed the least energy during the reaction with obtaining the highest energy values in the solid products [7,8]. Due to this, torrefaction attracted increasingly attention [9]. Generally, the torrefaction process involves four stages [1]. At first, biomass is heated to the drying temperature (<105 °C) and the free water inside the biomass evaporates. As the temperature continuously rises (<200 °C), the bound water is removed accompanied by minor mass loss caused by the slightly degradation of the organics. Then, it is the torrefaction process operated at a desired temperature (200~300 °C) involving the decomposition of hemicellulose, cellulose and partial lignin [10]. Finally, the torrefied biomass is cooled to room temperature. It is widely accepted that the properties of torrefied biomass, such as HHV, bulk density, energy density and grindability etc., are upgraded by reducing the water and oxygen content [11,12,13].

Because of the upgraded properties, torrefied biomass can easily replace coal in combustion or be co-fired with coal for electric power generation and heat supply [14,15]. Investigation of kinetics is the fundament to reveal the combustion mechanisms and to provide references for designing industrial facilities. Thermogravimetric analysis (TGA) has been proved to be an effective method to understand the combustion mechanism of solid fuels [16]. Ignition temperature (*T*_i_), burnout temperature (*T*_p_) etc. are commonly used parameters for assessing the combustion performance [17,18]. Nyakuma et al. [19] found that the torrefied pellets of oil palm empty fruit bunches were less thermally reactive compared to the raw pellet. In addition, few studies researched the kinetic mechanisms of torrefied biomass. Torrefaction was found to increase the activation energy (*E*_a_) and enhance the thermal stability of oil palm wastes [16], whereas it decreased the *E*_a_ of the spent coffee grounds and pigeon pea stalk [20,21]. However, Gürel et al. [22] reported that the torrefied biomass fuels exhibited the same qualitative behavior as the raw fuels. It can be seen that there are still conflicts in the results in the existing literature, and there is still confusion about the kinetic mechanisms of torrefied biomass combustion.

In addition to the kinetics, gas pollutants emission is another focus for torrefied biomass utilization as solid fuel. Ma et al. [23] found that torrefaction effectively decreased CO, NO and SO_2_ emissions during combustion of municipal wastes. For Bamboo residues, torrefaction reduced NH_3_ and NO emissions [24]. Maxwell et al. [25] also found that the emission of NOx decreased for torrefied biomass combustion, despite of the increased fuel-N content. On the contrary, torrefaction increased the fuel-N contents and subsequently resulted in a higher NOx emissions for kenaf [26] and Grape pomace [27]. However, torrefaction of banana leaf reduced CO, CO_2_ and SO_2_ emissions potential than commercial hard coal, except for NOx [28]. It was reported that the conversions and emission factors of gas pollutants varied with biomass type [29,30].

As reviewed above, the upgradation on the properties of biomass after torrefaction was widely accepted. However, investigations on the kinetic mechanisms and gas pollutants emissions are still controversial. Due to this, more studies are still needed to complement information on torrefied biomass utilization as fuel. It is widely reported that torrefaction is far more sensitive to temperature than residence time and heating rate [21,31]. Thus, in this study, pine sawdust was traditionally torrefied and the properties of torrefied samples integrated with the combustion kinetic mechanism and the emissions were investigated by non-isothermal combustion and isothermal experiments. The results could provide complementary information for the design, optimization and modification of industrial facilities.

## 2. Materials and Methods

### 2.1. Materials

Pine sawdust (PS), a typical woody biomass was employed as sample in this study. Before experiments, the raw material was dried for 24 h at 105 °C and then pulverized. The particle size of 60~100 mesh was then sieved for subsequent torrefaction experiments and analysis.

### 2.2. Methods

#### 2.2.1. Torrefaction of PS

Torrefaction experiments were conducted in a horizontal tube furnace (Yifeng Electric Furnace Co., LTD, Shanghai, China). A total of 3 ± 0.01 g of the PS powder was weighted in each experiment. Then, the samples were lightly torrefied at 200 °C, mildly at 250 °C and severely at 300 °C in N_2_ atmosphere for 1 h, respectively. The purity of the N_2_ was 99.999% and the flow rate was 0.5 L/min. After experiments, the solid product was cooled to the room temperature with the protection of N_2_ and then weighted and collected for further characterization and experiments. The torrefied PS produced at 200 °C, 250 °C and 300 °C were denoted as PS200, PS250 and PS300, respectively.

#### 2.2.2. Characterization

To evaluate the evolution of chemical components after torrefaction, proximate and ultimate analysis for the raw and torrefied PS were investigated according to the methods described in our previous study [32]. HHV was also tested by a calorimeter bomb for the fuel purpose.

Mass yield and energy yield were calculated following Equations (1) and (2) [7], respectively.

(1) $$M=\frac{m_{1}}{m_{0}}\times 100\%$$

(2) $$E=M\times \frac{{HHV}_{1}}{{HHV}_{0}}\times 100\%$$

where *M* is the mass yield, wt.%; *m*_1_ and *m*_0_ are the mass of torrefied product and raw biomass, respectively, g; *E* is the energy yield, %; *HHV*_1_ and *HHV*_0_ are the high heating value of torrefied product and raw biomass, respectively, MJ/kg.

#### 2.2.3. Non-Isothermal Combustion

Non-isothermal combustion tests were performed using a TG analyzer (TGA 4000-SPECTRUM TWO, PerkinElmer, MA, USA) in air atmosphere to investigate the combustion behaviors of the raw and torrefied PS. 10 ± 0.1 mg of the samples were weighted and then loaded into an alumina ceramic crucible during each run. The flow rate of air was 100 mL/min. The temperature programming was set from room temperature to 800 °C with a heating rate of 10 °C/min.

Ignition temperature (*T*_i_) and burnout temperature (*T*_b_) were calculated to evaluate the thermal stability of the solid fuel. *T*_i_ was determined by TG/DTG plots using the intersection method (IM) [33] and *T*_b_ was the point where the weight loss rate was lower than 1 wt.%/min [17]. *DTG*_max_ was the highest weight loss rate in each stage and peak temperature (*T*_p_) was the temperature at this point, both of which can be directly determined by DTG curves [26].

To further assess the combustion performance of the solid fuel, flammability index (*C*_i_, wt.% min^−1^ °C^−2^) and comprehensive combustion index (*S*, wt.%^2^ min^−2^ °C^−3^) were calculated following equation (3~4) [28,34]. Especially, *S* is a comprehensive parameter integrates the ignition and burnout characteristics of the solid fuel [20]. A higher *S* indicates better combustion reactivity [24].

(3) $$C_{i}=\frac{{DTG}_{\max}}{T_{i}^{2}}$$

(4) $$S=\frac{{DTG}_{\max}\times {DTG}_{mean}}{T_{i}^{2}\times T_{b}}$$

where *DTG*_mean_ is the average weight loss rate from ignition to burnout temperature, wt.%/min.

#### 2.2.4. Kinetic Analysis

Kinetics mechanism provide valuable information for designing and optimizing the combustion facilities. In this study, model fitting methods of an integral method (Coats–Redfern method, CR) [35,36,37] and differential method (Freeman–Carroll, FC) [16,38] were employed for calculation of kinetics parameters using TGA data. The conversion rates of all reactions can be described by Arrhenius expression:

(5) $$\frac{d\alpha }{dt}=kf(\alpha )$$

(6) $$k=A\exp(-\frac{E_{a}}{RT})$$

where *α* is the conversion degree defined as (*m*_0_ − *m*_t_)/(*m*_0_ − *m*_f_); *m*_0_ is the initial mass of the sample, g; *m_t_* is the mass of the sample at time *t*, g; *m_f_* is the final mass of the sample, g; *E*_a_ is the apparent activation energy (kJ/mol); *A* is the pre-exponential factor (s^−1^); *R* is the universal gas constant; *T* is the temperature (K). A larger *E*_a_ indicates that the reaction is more difficult to process while a larger *A* indicates the more active the reaction [36].

Then, the conversion rate can be rewritten as the following equation:

(7) $$\frac{d\alpha }{dt}=A\cdot e^{\frac{-E_{R}}{RT}}\cdot f(\alpha )$$

In this study, the TGA is non-isothermal process but the heating rate is constant, so Equation (7) can be modified considering the heating rate *β* as *dT* = *β*⋅*dt*:

(8) $$\frac{d\alpha }{dT}=\frac{A}{\beta }\cdot e^{\frac{-E_{R}}{RT}}\cdot f(\alpha )$$

The most commonly used model for *f*(*α*) is defined as:

(9) $$f(\alpha )={\left(1-\alpha \right)}^{n}$$

To simplify the kinetic calculations, biomass combustion reactions were often considered as first order reaction in many literatures. However, the combustion process is actually complicated involving homogeneous and heterogeneous reactions. Therefore, different *f*(α) models were used in this study to calculate *E*_a_ and *A*.

For CR method:

(10) $$g(\alpha )=\int \frac{1}{f(\alpha )}d\alpha =\frac{A}{\beta }\int e^{\frac{-E_{a}}{RT}}dT$$

Integrating and then taking logarithms at both sides, Equation (10) becomes:

(11) $$\ln\frac{g(\alpha )}{T^{2}}=\ln\left[\frac{A}{\beta }\frac{R}{E_{a}}\left(1-\frac{2RT}{E_{a}}\right)\right]-\frac{E_{a}}{RT}$$

Here, (1 − 2*RT*/*E*_a_) can be approximately equal to 1 because *RT*/*E*_a_ is far smaller than 1. Then, a line can be obtained with a slope and *y*-intercept using for the calculation of *E*_a_ and *A*, respectively.

For FC method, taking logarithms at both sides of Equation (8):

(12) $$\ln\left(\frac{d\alpha }{dT}\right)=\ln\frac{A}{\beta }-\frac{E_{a}}{RT}+n\ln\left(1-\alpha \right)$$

Using interpolation method, Equation (12) becomes:

(13) $$\frac{\Delta \ln\left(\frac{d\alpha }{dT}\right)}{\Delta \ln(1-\alpha )}=n-E_{a}\frac{\Delta T^{-1}}{R\Delta \ln(1-\alpha )}$$

Then, a regression line can be obtained with a slope and *y*-intercept directly corresponding to *E*_a_ and *n*, respectively. After defining *E*_a_ and *n*, *A* can be obtained from Equation (12).

#### 2.2.5. Isothermal Combustion

Isothermal combustion experiments were carried out in the horizontal tube furnace at 800 °C to estimate the emissions. 50 ± 0.1 mg samples were employed in each test. The flow rate of air was 50 mL/min. CO_2_, CO and NO concentrations were on-line tested by a flue gas analyzer (NOVAplus, MRU Instruments, Inc., Neckarsulm, Germany). Each experiment was repeated three times and the average value of conversion rate was calculated.

## 3. Results and Discussion

### 3.1. Properties

#### 3.1.1. Proximate and Ultimate Analysis

**Figure 1:**
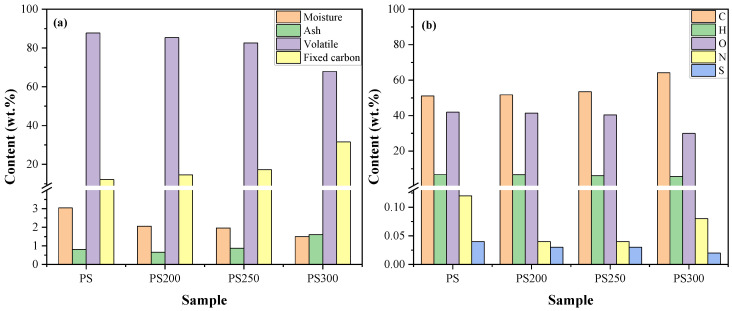
Proximate (**a**) and ultimate (**b**) analysis results.

The results of proximate and ultimate analysis of the raw and torrefied PS are shown in Figure 1 and the detailed information was given in Table S1 in the Supplementary Material. The contents of volatile and moisture decreased while those of ash and fixed carbon increased after torrefaction. This should be attributed to the evaporation and degradation of organics during torrefaction. It was reported that the stability of the organics in biomass followed in the order of hemicellulose decomposed at around 180 °C < cellulose at around 200 °C < lignin at approximately 300 °C [11,12]. Due to this, the degradation of the organics was more violent at 300 °C, leading to quite a significant reduction of volatile content and an increase of fixed carbon content in PS300 compared with that of PS200 and PS250, accompanied by the enrichment of ash components in the solid product.

The changes of the CHO contents were negligible when PS was lightly and mildly torrefied, while remarkable changes were observed at 300 °C. This means that the degradation of organics was much violent at 300 °C. At this temperature, a large amount of CHO elements is released in the form of CO_2_, CO and H_2_O via dihydrogen and dicarboxylic reactions of cellulose, hemicellulose and partial lignin [11,12,39]. The N content in PS, PS200, PS250 and PS300 was 0.12 wt.%, 0.04 wt.%, 0.04 wt.% and 0.08 wt.%, respectively. Thus, it can be calculated that 68.58 wt.%, 72.09 wt.% and 64.82 wt.% of the fuel-N was released in the torrefaction process, respectively. In general, the N and S contents decreased after torrefaction which might be beneficial to the reduction of NOx and SO_2_ pollutants in the consequently combustion process. Additionally, the S content in the raw and torrefied PS was tiny (less than 0.04 wt.%) that can be ignored.

**Figure 2:**
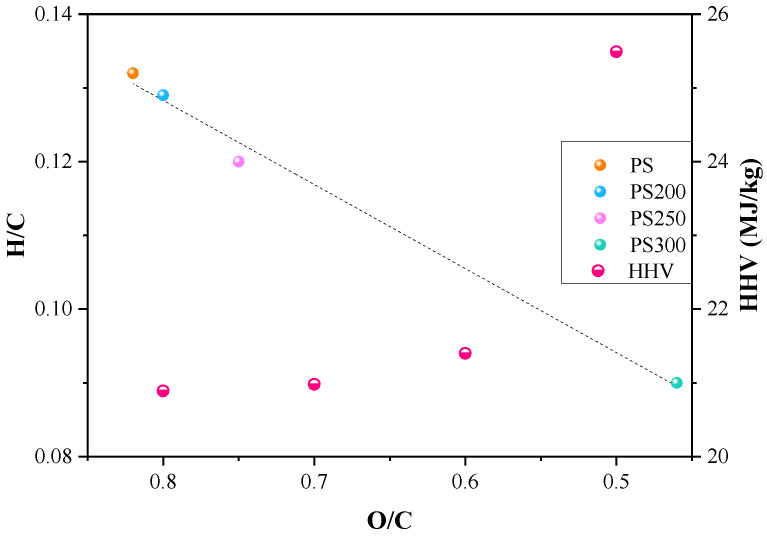
Van Krevelen Plot and HHV variation of raw and torrefied PS.

A liner relationship between O/C and H/C was found in the Van Krevelen Plot as shown in Figure 2. As temperature increased, both O/C and H/C ratios decreased, indicating a higher energy density for solid fuel [1]. The raw PS has an O/C ratio of 0.82 and H/C ratio of 0.132. After torrefaction, the values of O/C and H/C ratio decreased to 0.8 and 0.129, 0.75 and 0.12, 0.46 and 0.09 for PS200, PS250 and PS300, respectively, implying the removal of O and H elements during torrefaction caused by the dehydration, deoxygenation and dehydrogenation reactions of organics [21]. This is highly in line with the results of proximate and element analysis. Further, the reduction of O/C and H/C ratios also led to an increase in the HHV of the solids, especially for PS300. The value of HHV was 20.89 MJ/kg for PS, while it reached to 25.49 MJ/kg for PS300, which is comparable to that of coal (25–35 MJ/kg) [1].

#### 3.1.2. Mass and Energy Yields

**Figure 3:**
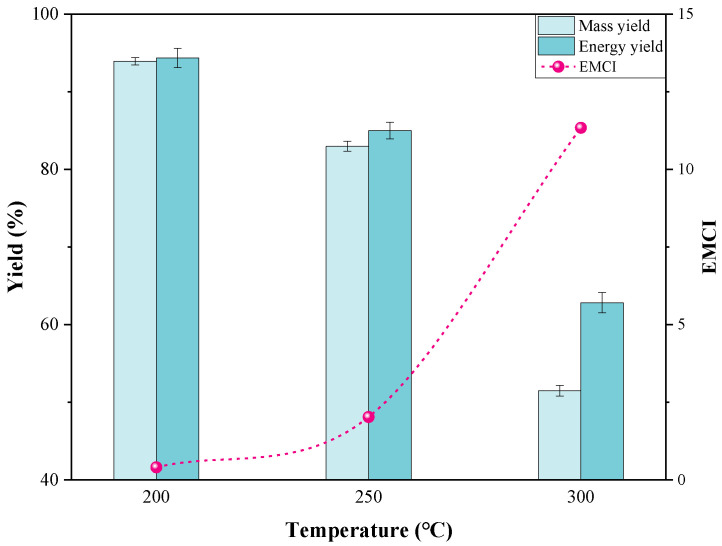
Mass yield, energy yield and EMCI versus temperature.

As shown in Figure 3, the mass yield decreased with rising temperature. When torrefied at 200 °C, the weight loss of the solids was inconspicuous and the mass yield was 93.9 wt.%. This was attributed to the precipitation of a small amount of water, including evaporation and the slightly dehydration reaction of hemicellulose with poor stability [32]. Remarkable weight loss was observed at 300 °C with a mass yield of 51.5 wt.%, indicating that the degradation of hemicellulose, cellulose and lignin was much violent at a higher temperature [40]. The energy yield versus temperature showed the same tendency as the mass yield. However, the energy yield was always higher than the mass yield in any case. This is quite consistent with previous studies [12,41].

EMCI, an energy-mass co-benefit index that comprehensively considers the mass and energy yields [42] was evaluated. A larger EMCI indicates a higher energy density and a less volume of the solids, which are beneficial to increase processing efficiency and to facilitate transport [43]. The values of EMCI are simultaneously provided in Figure 1. At 200 °C and 250 °C, it was 0.40 and 2.03, respectively, while it reached to 11.34 at 300 °C. From this perspective, 300 °C was the optimal temperature for PS torrefaction.

### 3.2. Non-Isothermal Combustion

**Figure 4:**
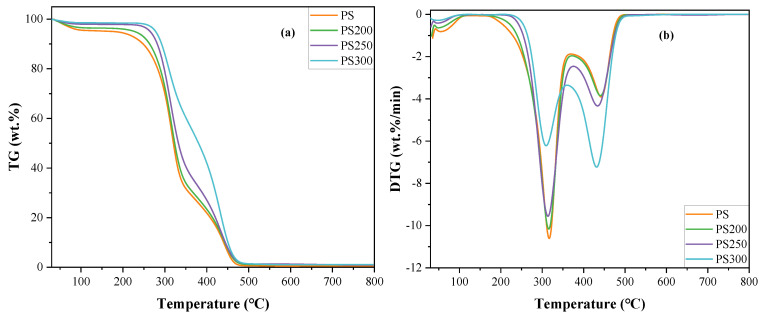
TG and DTG curves for the raw and torrefied PS combustion at 10 °C/min. (**a**) TG; (**b**) DTG.

Figure 4 illustrated the non-isothermal profiles for the combustion of the raw and torrefied PS. As shown in Figure 4a, the TG curves for PS200 and PS250 was not significantly affected by torrefaction. However, it was quite different for PS300, implying that the properties of PS were not greatly changed until the torrefaction temperature reached to 300 °C. In addition, there were three peaks for weight loss rate in the DTG curves, indicating that the combustion process could be divided into three stages: the drying stage (stage I), devolatilization and combustion stage (stage II), and char combustion stage (stage III). The mineral decomposition stage (stage IV) reported by Ullah et al. [44] was not observed in the profiles due to the negligible ash content in the raw and torrefied PS.

The drying stage occurred at temperatures below 150 °C during which the weight loss was usually assigned to the evaporation of absorbed moisture [17,28]. The maximum weight loss rate was 0.8 wt.%/min, 0.63 wt.%/min, 0.4 wt.%/min and 0.27 wt.%/min observed at 60 °C, 46.7 °C, 53.1°C and 54.1 °C for PS, PS200, PS250 and PS300, respectively. The decrease in the maximum weight loss rate indicated the reduction of moisture content in the torrefied PS.

The temperature range for devolatilization and combustion was from 150 °C to 369.1 °C, 371.7 °C, 375.4 °C and 360.5 °C for PS, PS200, PS250 and PS300, respectively. In this stage, the volatiles released due to the devolatilization and depolymerization of hemicellulose, cellulose and lignin, and then oxidized accompanied by the formation of the char matrix [28]. Then, it was the char combustion stage, which continued to approximately 500 °C.

Table 1 shows the combustion characteristics and indices of the raw and torrefied PS. Torrefaction increased the ignition temperature (*T*_i_) from 272.9 °C for PS to 289.6 for PS300 in virtue of the breakdown of hemicellulose and retaining most of the cellulose and lignin in torrefied PS [45]. This led to the delay of the devolatilization and combustion. Alves et al. [28] also found that the ignition temperatures for torrefied banana leaf waste were 8~14 °C higher than that of the original sample due to the lower values of volatile, hydrogen and oxygen contents. The burnout temperatures (*T*_b_) for torrefied PS were also higher than the raw PS, indicating a longer combustion process for torrefied PS because of the increase of the lignin content [17].

Compared to PS, the peak temperatures (*T*_p_) of both volatile and char combustion for torrefied PS samples shifted to lower values, implying the upgradation of the reactivity of the solid fuels after torrefaction. The maximum weight loss rate (*DTG*_max_) and the weight loss (*W*_L_) of the volatile combustion decreased while that of the char combustion increased. Especially for PS300, the *DTG*_max_ for the overall combustion process even shifted from volatile combustion to char combustion stage. This should be attributed to the reduction of volatile content and the increase of the fixed carbon content [19].

The flammability index (*C*) decreased with rising torrefaction temperature because torrefaction reduced the flammable components (mainly hemicellulose and cellulose) in the solid fuels [17]. The comprehensive combustion index (*S*) was not affected by light torrefaction, while it slightly decreased after mild and severe torrefaction. The values of *S* for torrefied poultry litter at 250–280 °C were also found to be lower than that of the raw sample [18]. It was reported that *S* was positively affected by hemicellulose while negatively correlated with lignin [46]. Duman et al. [47] researched the combustion behavior of torrefied olive tree pruning and vineyard pruning and came to the conclusion that torrefied biomass had lower *S* values compared to the raw samples. They suggested that *S* was affected by several factors, such as the number of active sites, oxygen content, inorganic content, etc. However, the decrease of *S* in this study was tiny that could be ignored.

### 3.3. Kinetic Mechanism

According to the DTG profiles in Figure 4, the combustion process of the raw and torrefied PS from *T*_i_ to *T*_p_ could be approximately assigned to two independent reactions involving volatile combustion and char combustion. Thus, the kinetic parameters *E*_a_ and *A* for each stage were determined by both CR and FC methods. To reveal the combustion reaction mechanisms, various function models (Table S2 in the Supplementary Materials) were used. The results corresponding to the best correlation coefficient (R^2^) for the two methods are shown in Table 2.

As shown in Table 2, all of the raw and torrefied PS presented good fitting results for both methods. The combustion reactions of volatiles followed degradation mechanisms while diffusion mechanisms were followed for the char combustion process. Castells et al. [16] also reported that degradation was followed by a diffusion reaction during combustion torrefied empty fruit bunches, palm kernel shell and palm mesocarp fiber, which are different parts of the palm oil tree. Thus, the dominate mechanism for the raw and torrefied PS combustion was the degradation reaction.

For both methods, the *E*_a_ values of the volatile combustion stage were much larger than those of the char combustion stage, indicating that the combustion process is dominated by devolatization [26]. This is because the release of volatiles from the sample needed energy. However, the ignition of the chars was accelerated with the support of the heat generated by volatile combustion, so less additional energy was needed for the char combustion. This was also reported by Xin et al. [41] Therefore, attention should be paid to the initial combustion stage of the torrefied biomass. This was consistent with previous study [36].

There were differences on the tendency of *E*a values for volatile combustion between the CR and the FC method. For the CR method, it increased after light and mild torrefaction with a maximum value of 257.52 kJ/mol for PS250, and then decreased to 165.24 kJ/mol for PS300. This might be attributed to the destroyed structure of the solid at a higher temperature, which brought about decreases in kinetic parameters [16]. Ma et al. [23] also observed the highest value of *E*a for TBP250 using CR method and a decline at above 250 °C. While for the FC method, it invariably decreased with the rising torrefaction temperature. However, for both methods, PS300 had the lowest *E*a value, indicating that PS300 had better combustibility and reactivity. This might be attributed to the more developed pore structures under severe torrefaction temperature. The significantly destroyed morphological structure of a torrefied biomass at 300 °C has been reported in the other literature [19,24], which might enhance the diffusion of volatiles into oxygen and then bring about decreases in kinetic parameters [23]. Nevertheless, the *E*a value of the char combustion stage gradually increased with rising torrefaction temperature for both CR and FR methods. Bach et al. [48] also reported an increase in kinetic parameters of char after torrefaction of Stem wood at 275 °C. Higher torrefaction temperatures were indicated for the reduction of volatile content in torrefied PS and the decrease of devolatilization during combustion, which subsequently led to less heat generation by volatile combustion. Thus, more additional energy was needed for the char combustion, and the *E*a values increased.

### 3.4. Emissions

**Figure 5:**
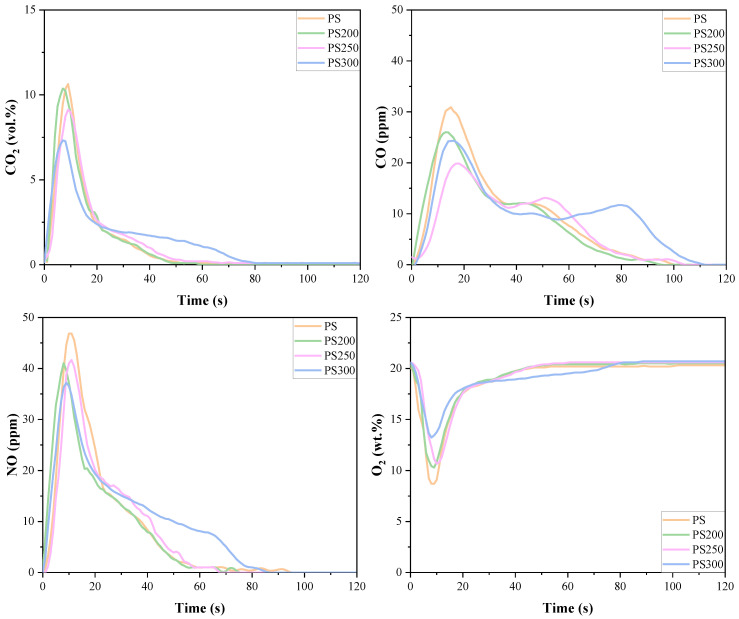
CO_2_, CO and NO emissions and O_2_ consumption of isothermal combustion.

The emission profiles of CO_2_, CO and NO during isothermal combustion are shown in Figure 5. Meanwhile, the consumption profiles of O_2_ were also presented to provide sufficient information for analysis and discussion. It is obvious that the isothermal combustion process at 800 °C could be divided into two stages involving volatile combustion in the first ~20 s and char combustion lasted for the rest time.

When the combustion began, the O_2_ concentrations sharply decreased within 10 s due to the rapid oxidation of released volatiles. This was also responsible for the delayed formation of CO. As torrefaction temperature increased, the consumption of O_2_ caused by volatile oxidation decreased, owing to the reduction of volatile content in the torrefied PS, and for this reason, the concentration of CO_2_ decreased. However, a different trend was observed for CO emission. The concentration of CO firstly decreased and then increased with a minimum value for PS250. The increase in CO concentration for PS300 might be attributed to the more developed pore structures, which promoted the release of volatiles and then brought about incomplete combustion. Hu et al. [24] revealed that the morphological structure of bamboo residues was significantly changed after being torrefied at 300 °C, with completely broken fibers and superficially porous structures which enhanced the gas volatilization. Nyakuma et al. [19] also observed a dense network of macropores rather than the microporous and mesoporous fibers for torrefied pellets at 300 °C, which led to a significantly different fuel property. This might also be responsible for the reduction of NO concentration with rising torrefaction temperature because the developed pore structure of the char matrix could provide more active sites for NO reduction by CO and char. Another possible reason for NO reduction is the shift of unstable ammonia-N in biomass to heterocyclic-N which were tightly bound to the char matrix [32]. Furthermore, the time point for the peak value of O_2_ consumption was slightly delayed for PS200 and PS250 and then brought forward for PS300 in this stage, indicating that the combustibility of PS300 was much better than others. This was quite consistent with the results obtained from kinetics calculation that the *E*_a_ for PS300 was much smaller than that of other samples.

For the char combustion stage, the consumption of O_2_ was more pronounced for PS300 due to the significant increase in fixed carbon content. Meanwhile, the emissions of CO_2_ and CO for PS300 were notably intensified, meaning that the emissions of pollutants shifted from the volatile combustion to the char combustion stage. This was consistent with the special properties of PS300 and its combustion characteristics observed in TG/DTG profiles. Furthermore, NO concentration also increased with rising torrefaction temperature because the shift of ammonia-N to heterocyclic-N increased the char-N content [24].

**Figure 6:**
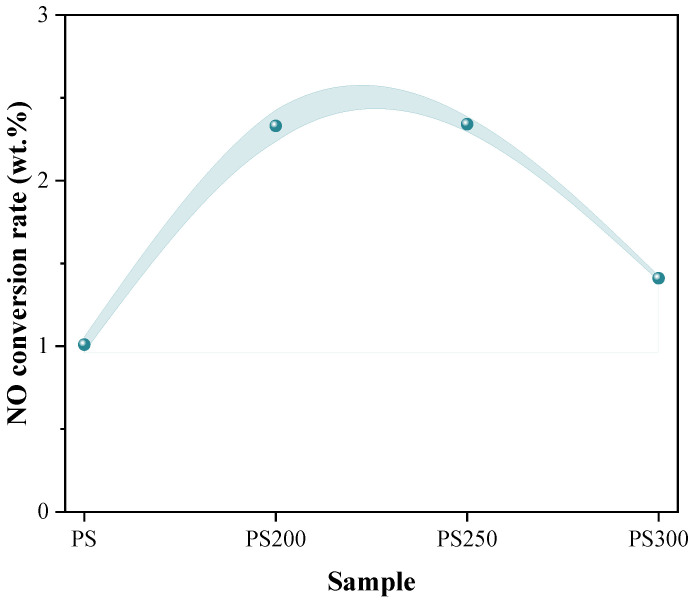
Conversion rate of fuel-N to NO during combustion.

To better understand the emission of NO in the overall combustion process, the conversion rate of fuel-N to NO was calculated by Equation (4) given in our previous study [49], and the results are shown in Figure 6. The conversion of fuel-C to CO was tiny (<0.01 wt.%), so it was not discussed here. It can be seen that the conversion rates of fuel-N to NO for torrefied PS combustion were slightly higher than that of the raw PS. With torrefaction temperature rising, the conversion rate firstly increased and then decreased. This was contrary to the results obtained by Lee et al. [26]. Maxwell et al. [25] reported a linear relationship between NOx emission and nitrogen content. However, in this study, the conversion was inversely correlated to the fuel-N content which was presented in Figure 1b. Yanik et al. [50] also found that the NO emission from the combustion of torrefied poultry litter was higher although the fuel-N content was lower than the raw poultry litter. This should be explained by the reduction of NOx precursors, while the increase of N_2_ formation with rising fuel-N content during pyrolysis [51] and subsequently less fuel-N was oxidized into NO.

## 4. Conclusions

This study aimed to provide complementary information for torrefied biomass utilization as solid fuels, regarding not only the properties but also the combustion kinetic mechanisms and emissions. In view of this, torrefied pine sawdust (PS) was produced and both isothermal and non-isothermal combustion experiments were conducted. Results showed that torrefaction significantly improved the quality of PS, which are a benefit to its fuel utilization. Further, degradation mechanisms for volatile combustion and diffusion mechanisms for the char combustion were determined by using both Coats–Redfern (CR) and Freeman–Carroll (FC) kinetic methods. Moreover, during the isothermal combustion, the emissions of CO_2_, CO and NO decreased for volatile, while they increased for char. Additionally, the conversion of fuel-N to NO was found to be inversely related to the nitrogen content in the samples. In conclusion, this study provided complementary information for understanding biomass torrefaction and designing of industrial facilities.

## Figures and Tables

**Table 1:** Combustion characteristics and indices of the raw and torrefied PS.

Sample	*T_i_* (°C)	*T_b_* (°C)	Volatile Combustion			Char Combustion			*C* × 10^4^ (wt.%/min/°C^2^)	*S* × 10^7^ (wt.%^2^/min^2^/°C^3^)
			T_p_ (°C)	DTG_max_ (wt.%/min)	WL (wt.%)	T_p_ (°C)	DTG_max_ (wt.%/min)	WL (wt.%)		
PS	272.9	470.8	316.4	−10.60	55.62	444.0	−3.89	35.85	1.36	0.12
PS200	277.5	473.6	315.4	−10.16	41.80	442.6	−3.83	48.02	1.32	0.12
PS250	280.5	474.6	313.9	−9.55	33.83	433.7	−4.33	51.66	1.21	0.11
PS300	289.6	487.3	309.5	−6.21	19.93	431.4	−7.23	57.29	0.93	0.10

**Table 2:** Kinetic parameters of the raw and torrefied PS.

	Sample	Temp. Interval (°C)	Model Function	R^2^	*E*a (kJ/mol)	A (s^−1^)
CR method	PS	272.9–369.1	g5	0.98086	175.2788	6.1565 × 10^12^
		369.1–470.8	D1	0.96173	14.9932215	3.6378 × 10^−4^
	PS200	277.5–371.7	g5	0.98649	171.834257	2.3500 × 10^12^
		371.7–473.6	D1	0.97575	16.3831073	4.9355 × 10^−4^
	PS250	280.5–375.4	g8	0.993	257.522588	2.1745 × 10^20^
		375.4–474.6	D1	0.98751	22.9869979	2.0432 × 10^−3^
	PS300	289.6–360.5	g8	0.99449	165.240647	1.9976 × 10^11^
		360.5–487.3	D2	0.98881	67.5765155	7.5432 × 10^0^
FC method	PS	272.9–369.1	g8	0.95713	291.9849953	8.5326 × 10^26^
		369.1–470.8	D1	0.97589	5.630441084	40.6876698
	PS200	277.5–371.7	g8	0.96829	281.847244	7.5434 × 10^25^
		371.7–473.6	D1	0.98257	5.902389863	42.4380384
	PS250	280.5–375.4	g8	0.97924	252.1634035	6.8384 × 10^22^
		375.4–474.6	D1	0.99073	7.120495037	51.7821292
	PS300	289.6–360.5	g8	0.98088	150.2658078	9.741 × 10^12^
		360.5–487.3	D1	0.98206	10.64739676	91.2940023

## Data Availability

Not available.

## Supplementary Materials

The following supporting information can be downloaded at: https://www.mdpi.com/article/10.3390/ma15238650/s1, Table S1: Function models used in the kinetics calculations; Table S2: Function models used in the kinetics calculations.
