# Supplementary material for: Kinetic Mechanisms and Emissions Investigation of Torrefied Pine Sawdust Utilized as Solid Fuel by Isothermal and Non-Isothermal Experiments
Source: Materials (Basel). 2022 Dec 4;15(23):8650. doi: 10.3390/ma15238650 (PMC9737359; doi:10.3390/ma15238650)
Supplement: Supplementary file 1 [file materials-15-08650-s001.zip › materials-2023456-supplementary.pdf]

Supplementary Materials

Kinetic Mechanisms and Emissions Investigation of Torrefied Pine Sawdust Utilized as Solid Fuel by Isothermal and Non-Isothermal Experiments

Xiaorui Liu [1], Dong Li [1], Jiamin Yang [1] and Longji Yuan [2,*]

[1] School of Mine, China University of Mining and Technology, Xuzhou 221116, China
[2] School of Low-Carbon Energy and Power Engineering, China University of Mining and Technology, Xuzhou 221116, China
* Correspondence: yuanlongji@cumt.edu.cn

Table S1. HHV, proximate and ultimate analysis results of the raw and torrefied PS.

Property	PS	PS200	PS250	PS300
HHV, MJ/kg	20.89	20.98	21.4	25.49
Proximate analysis, ad, wt.%				
Moisture	3.04	2.06	1.96	1.5
Ash	0.66	0.81	0.87	1.6
Fixed carbon	12.13	14.55	17.24	31.56
Volatile	87.77	85.36	82.61	67.92
Ultimate analysis, ad, wt.%				
C	51.16	51.73	53.47	64.24
H	6.74	6.69	6.18	5.67
O	41.94	41.4	40.38	29.98
N	0.12	0.04	0.04	0.08
S	0.04	0.03	0.03	0.02

Table S2. Function models used in the kinetics calculations.

1	Mechanism	Symbol	f (α)	g(α)
Order of reaction				
1	First-order	g_1	$1-\alpha$	$-\ln(1-\alpha)$
2	Second-order	g_2	$(1-\alpha)^2$	$(1-\alpha)^{-1}-1$
3	Third-order	g_3	$(1-\alpha)^3$	$[(1-\alpha)^{-2}-1]/2$
n	n-order	g_n	$(1-\alpha)^n$	$[(1-\alpha)^{-(n-1)}-1]/(n-1)$
Diffusion				
4	One-way transport	D_1	0.5α	α^2
5	Two-way transport	D_2	$[-\ln(1-\alpha)]^{-1}$	$(1-\alpha)\ln(1-\alpha)+\alpha$
6	Three-way transport	D_3	$1.5(1-\alpha)^{2/3}[1-(1-\alpha)^{1/3}]^{-1}$	$[1-(1-\alpha)^{1/3}]^2$
7	Ginstling-Brounshtein equation	D_4	$1.5[(1-\alpha)^{-1/3}-1]^{-1}$	$(1-2\alpha/3)-(1-\alpha)^{2/3}$
Limiting surface reaction between both phases				
8	Two dimensions	R_2	$2(1-\alpha)^{1/2}$	$1-(1-\alpha)^{1/2}$
9	Three dimensions	R_3	$3(1-\alpha)^{2/3}$	$1-(1-\alpha)^{1/3}$
Random nucleation and nuclei growth				
10	one-dimensional	A_1	$(1-\alpha)$	$-\ln(1-\alpha)$
11	Two-dimensional	A_2	$2(1-\alpha)[-\ln(1-\alpha)]^{1/2}$	$[-\ln(1-\alpha)]^{1/2}$
12	Three-dimensional	A_3	$3(1-\alpha)[-\ln(1-\alpha)]^{2/3}$	$[-\ln(1-\alpha)]^{1/3}$
